# Genomic control of inflammation in experimental atopic dermatitis

**DOI:** 10.1038/s41598-022-23042-x

**Published:** 2022-11-07

**Authors:** Yan Liu, Jozef Zienkiewicz, Huan Qiao, Katherine N. Gibson-Corley, Kelli L. Boyd, Ruth Ann Veach, Jacek Hawiger

**Affiliations:** 1 Department of Medicine, Division of Allergy, Pulmonary and Critical Care Medicine, Vanderbilt University School of Medicine, Vanderbilt University Medical Center, 21St Avenue South, T-1218, MCN, Nashville, TN 37232 USA; 2 Department of Veterans Affairs, Tennessee Valley Health Care System, Nashville, TN USA; 3 Department of Pathology, Microbiology and Immunology, Vanderbilt University School of Medicine, Nashville, TN USA; 4 Department of Medicine, Division of Nephrology, Vanderbilt University School of Medicine, Nashville, TN USA; 5 Department of Molecular Physiology and Biophysics, Vanderbilt University School of Medicine, Nashville, TN USA

**Keywords:** Inflammation, Peptide delivery

## Abstract

Atopic Dermatitis (AD) or eczema, a recurrent allergic inflammation of the skin, afflicts 10–20% of children and 5% adults of all racial and ethnic groups globally. We report a new topical treatment of AD by a Nuclear Transport Checkpoint Inhibitor (NTCI), which targets two nuclear transport shuttles, importin α5 and importin β1. In the preclinical model of AD, induced by the active vitamin D_3_ analog MC903 (calcipotriol), NTCI suppressed the expression of keratinocyte-derived cytokine, Thymic Stromal Lymphopoietin (TSLP), the key gene in AD development. Moreover, the genes encoding mediators of T_H2_ response, IL-4 and its receptor IL-4Rα were also silenced together with the genes encoding cytokines IL-1β, IL-6, IL-13, IL-23α, IL-33, IFN-γ, GM-CSF, VEGF A, the chemokines RANTES and IL-8, and intracellular signal transducers COX-2 and iNOS. Consequently, NTCI suppressed skin infiltration by inflammatory cells (eosinophils, macrophages, and CD4 + T lymphocytes), and reduced MC903-evoked proliferation of Ki-67-positive cells. Thus, we highlight the mechanism of action and the potential utility of topical NTCI for treatment of AD undergoing Phase 1/2 clinical trial (AMTX-100 CF, NCT04313400).

## Introduction

Atopic Dermatitis (AD), also known as eczema, is the most common, recurrent inflammatory skin disorder afflicting an estimated 10–20% of children and 5% of adults in all racial and ethnic groups in the US and abroad^1,2^, with the highest incidence among African Americans^3^. Therefore, the impact of AD on the well-being of a diverse population is significant and indicative of a need for effective and safe therapies. AD, caused by allergic insults, is mediated by inflammation and manifested by intense itch, recurrent eczematous lesions, and a fluctuating course^4^.

Treatment of AD mediated by a plethora of genes encoding the mediators of skin inflammation poses a significant challenge. Currently used broad spectrum therapy with glucocorticoids (e.g., triamcinolone), and targeted therapies with monoclonal antibodies (e.g., dupilumab, tralokinumab) blocking IL-4 receptor, or small molecule inhibitors targeting intracellular signaling intermediates, such as Janus Kinases (JAKs; e.g., upadacitinib and abrocitinib), Phosphodiesterase 4 (PDE4; e.g., crisaborole), and calcineurin (e.g., tacrolimus, pimecrolimus), only partially inhibit inflammatory signaling responsible for AD development and persistence^2,5–7^. Targeting one or two mediators of skin inflammation by inhibitors of JAKs, PDE4, calcineurin, as well as the receptor for IL-4 and IL-13 by monoclonal antibodies, has advanced the treatment of AD and other skin inflammatory diseases^2^. However, other untargeted mediators contribute to the refractoriness or relapse of AD (see Suppl. Fig. S1). In turn, a broad-spectrum therapy with glucocorticoids through their binding to the cognate nuclear receptor reprograms the inflammatory regulome in the cell’s nucleus while simultaneously enhancing the expression of metabolic genes causing hyperglycemia, hyperlipidemia, osteoporosis, and skin atrophy^7,8^.

An alternative to glucocorticoids therapy is topical treatment with Nuclear Transport Checkpoint Inhibitor (NTCI). This anti-inflammatory cell-penetrating peptide targets two nuclear transport shuttles, cytoplasmic adaptor proteins termed importin α5 and importin β1^7,9,10^. Targeting these two proteins that comprise the nuclear transport checkpoint (see Suppl. Fig. S1) produces a broad-spectrum anti-inflammatory activity through silencing of at least 50 genes encoding mediators of inflammation and metabolism^7^. Their expression depends on the nuclear transport/translocation of an entire set of proinflammatory Stress-Responsive Transcription Factors (SRTFs), which include NF-κB, cFos/cJun, STAT1, and NFATs. Most recently, we found that the nuclear import of STAT3, the transcription factor involved in allergic inflammation, is inhibited by targeting importin α5 with NTCI in primary human keratinocytes derived from adult skin (unpublished data). In parallel, Metabolic Transcription Factors (MTFs) are also translocated to the cell’s nucleus. MTFs comprise Sterol Regulatory Elements-Binding Proteins (SREBPs) translocated solely by importin β1, and Carbohydrate Response Element-Binding Proteins (ChREBPs) shuttled either by importin α5 that recognizes the NLS “zip code” or importin β1 that binds to the Helix-Loop-Helix Leucine Zipper-binding site^7^ (see Suppl. Fig. S1). NTCI, the cSN50.1 peptide, stopped not only SRTFs and SREBPs but also ChREBPs on their path to nucleus by targeting the two components of the nuclear transport checkpoint, importin α5 and importin β1. Hence, the inflammatory regulome in the genome comprising the multiple genes that encode the mediators of allergic, autoimmune, microbial, and metabolic inflammation, cannot be activated^7^. We postulated that AD can be treated by NTCI through inhibition of nuclear signaling, thereby suppressing inflammatory injury to the skin.

The experimental model of AD caused by topical administration of MC903, the low calcemic analog of Vitamin D_3_, was introduced by Pierre Chambon’s group^11^ after calcipotriol was found to cause the signs of AD in clinical trials for the treatment of skin psoriasis^5^. MC903, applied topically to mouse ears, induces the characteristic signs of AD consisting of swelling, and T_H2_ type inflammation manifested in a dermal infiltrate. These cardinal criteria for MC903-induced AD were established by Gilhar and colleagues^12^. The pathognomonic features of AD were linked to epidermal keratinocytes expressing Thymic Stromal Lymphopoietin (TSLP). Its genetic ablation prevented AD-like skin changes upon challenge with MC903^13^. Moreover, TSLP role in promoting T_H2_ response and driving the pathogenesis is critical in the mechanism of AD^14^.

Keratinocytes are strategically positioned in the outermost layer of the skin, the epidermis^15^. They form a multilayered structure to maintain the functional integrity of the skin, which depends on three compartments within the epidermis: (i) the air–liquid barrier, (ii) the liquid–liquid barrier, and (iii) the immune defense barrier. Langerhans cells comprise the latter barrier. Their dendritic projections abut the keratinocytes, bridged by Tight Junctions (TJs), thereby demarcating the fluid microenvironment of the skin and favoring intercellular communication during the inflammatory response^15^. Keratinocytes, also named “cytokinocytes”, contribute to immune responses in skin^16^.

Keratinocytes sense and respond to proinflammatory insults by activating the signaling pathways to the nucleus, the site of the inflammatory regulome (see above)^7^. Therein, SRTFs and MTFs reprogram the inflammatory regulome and activate multiple genes that encode a myriad of inflammatory mediators. They encompass cytokines, chemokines, their receptors, cell adhesion molecules, as well as extracellular and intracellular signaling intermediates.

TSLP expressed by epidermal keratinocytes promotes T_H2_ response and drives the pathogenesis of AD^14^. Therefore, we aimed to establish whether NTCI would suppress TSLP and other genes involved in the allergic skin inflammation underlying AD.

We show that NTCI effectively reduces the cardinal signs of AD in an experimental model. To our knowledge, this study is the first preclinical proof of concept for the genomic control of AD by NTCI at the nuclear transport level. It can be extended to other inflammatory skin diseases. Its translational value is being evaluated in the ongoing Phase 1/2 Clinical Trial for mild to moderate AD (AMTX-100 CF, NCT04313400).

## Results

### Genomic control of experimental atopic dermatitis by a nuclear transport checkpoint inhibitor (NTCI)

**Figure 1:**
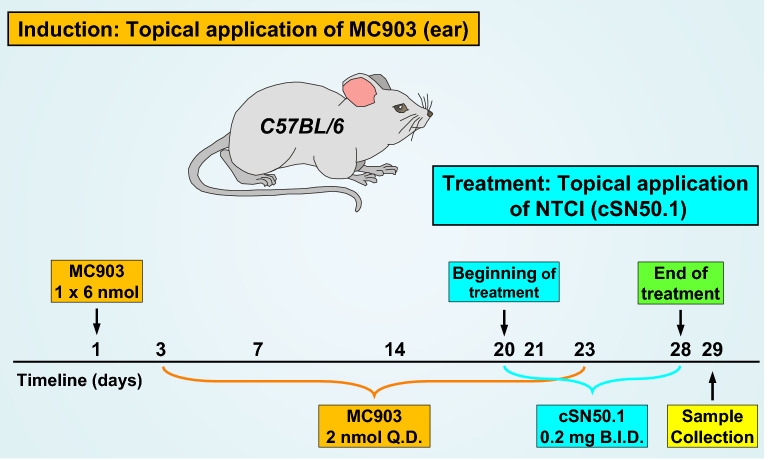
Graphic Depiction of Experimental Atopic Dermatitis and Treatment Protocol. Atopic dermatitis (AD)-like phenotype was induced in 8-week-old C57BL/6 female mice by topical application of MC903 (calcipotriol), an analog of vitamin D3. On day 1, an initial bolus dose of 6 nmoles of MC903 was applied topically to the right ear (3 nmoles on each side of the ear). On day 3, dosing once a day (Q.D.) with 2 nmoles of MC903 (1 nmole on each side of the right ear) was begun and continued for 3 weeks. On day 20, a 9-day twice-daily (B.I.D.) topical treatment with 66 nmoles NTCI (cSN50.1 peptide, 33 nmoles on each side of the right ear), or an equal volume of NTCI vehicle (10 µl saline on each side of the right ear) was initiated. Mice were weighed and ear thickness was measured every 2–4 days. Ear samples were collected on day 29 for gene expression and Immunohistochemistry (IHC) analyses. Mice in the Mock Control group were only subjected to the measurements of body weight and the ear thickness.

AD was induced by a topical application of MC903 to both sides of the right ear for 23 days (Fig. 1).

**Figure 2:**
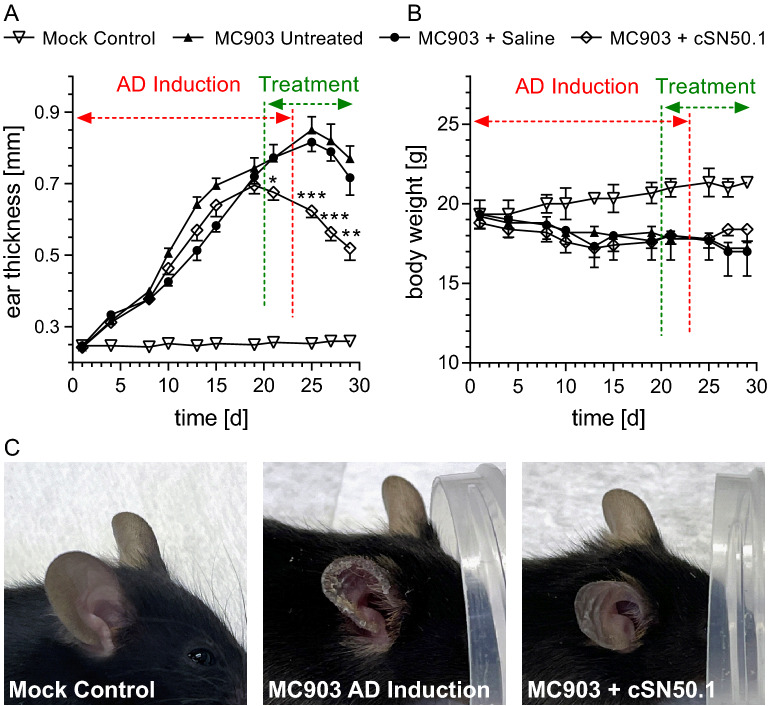
NTCI treatment reduces signs of MC903-induced Atopic Dermatitis (AD)-like phenotype manifested by swelling, redness, and scaling. Topical NTCI treatment accelerates healing in an experimental model of AD-like phenotype. Cardinal signs of inflamed skin, swelling (A), and redness and scaling (C) were significantly reduced by topical application of cSN50.1 peptide. (**A**) Ear thickness data is displayed as mean ± S.E.M. (n = 3 for Mock Control and n = 10 for all other conditions). Statistical significance of the difference between ear thickness in NTCI-treated mice (MC903 + cSN50.1) and untreated (MC903 Untreated) or saline-treated (MC903 + saline) groups was determined by two-way ANOVA using corrected Holm-Sidak test for multiple comparison, *p < 0.05, **p < 0.005, ***p < 0.0005. (**B**) Body weight was not significantly affected by MC903 challenge, NTCI, or by saline treatment albeit body weight gain was arrested in these groups. (**C**) Representative pictures of mice from the Mock Control group taken at the end of experiment and from the cSN50.1-treated group immediately before (MC903 AD Induction) and after NTCI treatment (MC903 + cSN50.1).

Topical treatment with an aqueous solution of NTCI, cell-penetrating cSN50.1 peptide, 200 µg (66 nmoles, administered twice daily to the right ear) was initiated on day 20 and continued for 9 days. The right ear of MC903-challenged mice became progressively swollen until treatment with NTCI significantly reduced skin edema, one of the chief signs of inflammation (Fig. 2A). Moreover, skin lesions, prominently displayed in the ear during the MC903 induction, were healed after 9 days of treatment with NTCI (Fig. 2C).

Thus, treatment with NTCI effectively reduced existing skin inflammatory signs, such as swelling, discoloration, and scaling. A troubling side effect of AD induction with MC903 is body weight loss. Notably, the animals treated with NTCI maintained their body weight (Fig. 2B), indicating a lack of NTCI’s general toxicity. This is consistent with the lack of apparent toxicity associated with long-term NTCI treatment in previous preclinical studies^10,17^. The lack of apparent toxicity of NTCI was also independently attested prior to the submission of the clinical trial protocol to the FDA (NCT04313400).

**Figure 3:**
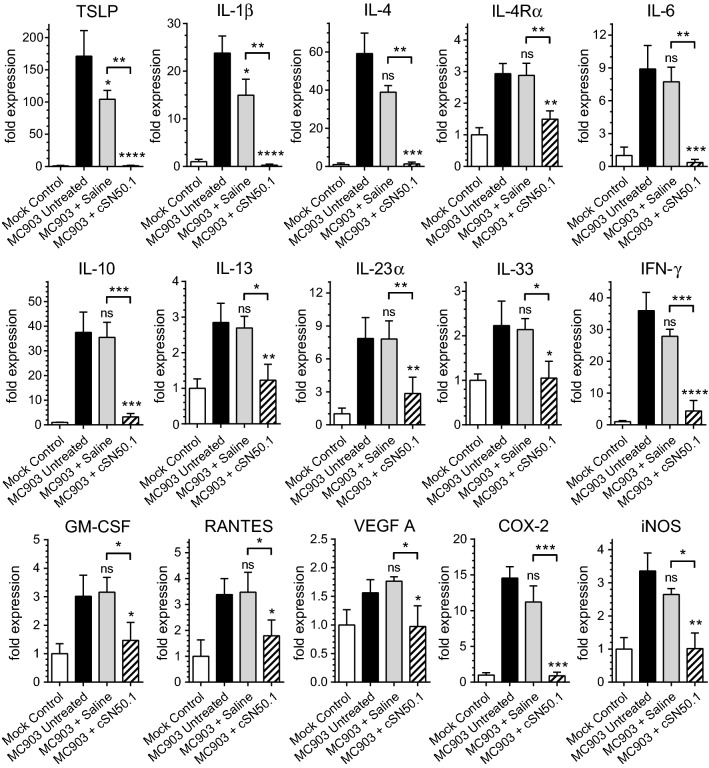
The expression of genes encoding the mediators of skin inflammation is suppressed by NTCI. The expression of genes encoding the mediators of allergic inflammation in the ear samples (see Methods and Fig. 1 for a depiction of AD-like phenotype induction and treatment protocol) was determined using a real-time quantitative reverse transcription PCR (qRT PCR). The relative levels of expression were established using Livak’s methods (2^−∆∆Ct^) with the 18S gene as a reference and the Mock Control (unchallenged ear) group as the calibrator. Data is presented as a mean + S.E.M. (n = 3 for Mock Control, n = 5 for all other conditions). A statistical analysis was performed using an ordinary one-way ANOVA with an uncorrected Fisher’s LSD test for a multiple comparison, *p < 0.05, **p < 0.005, ***p < 0.0005. The significance levels displayed over the bars of MC903 + Saline and MC903 + cSN50.1 columns represent the statistical difference of each compared to MC903 Untreated samples.

Gene expression profiling of skin obtained from animals challenged with MC903 and left untreated was compared to skin obtained from animals challenged with MC903 and treated with saline, and animals challenged with MC903 and treated with NTCI. Gene expression in skin obtained from mock control animals, which were not challenged with MC903, is also shown for comparison (Fig. 3).

The development of skin lesions in MC903-challenged ears was accompanied by the induction of genes encoding the mediators of allergic skin inflammation, including TSLP, the inducer of the T_H2_ response, and its mediators, IL-4, IL-4Rα, IL-13^14^ along with IL-6, IL-23α, IL-33, IFN-γ, and other cytokines, chemokines, growth factors, and intracellular signaling intermediates. TSLP activates Langerhans Cells, which initiate an adaptive T_H2_ response^14,18^, thereby demonstrating a central function of keratinocytes-derived TSLP in AD development. In addition, experimental AD was associated with increased expression of genes encoding the chemokine RANTES, as well as two intracellular signaling intermediates, COX-2, and iNOS, responsible for the synthesis of prostaglandins and nitric oxide (NO), respectively. The latter, together with VEGF A, are significant factors in the microvascular endothelial injury associated with skin inflammation manifested by swelling and itching^19^.

**Figure 4:**
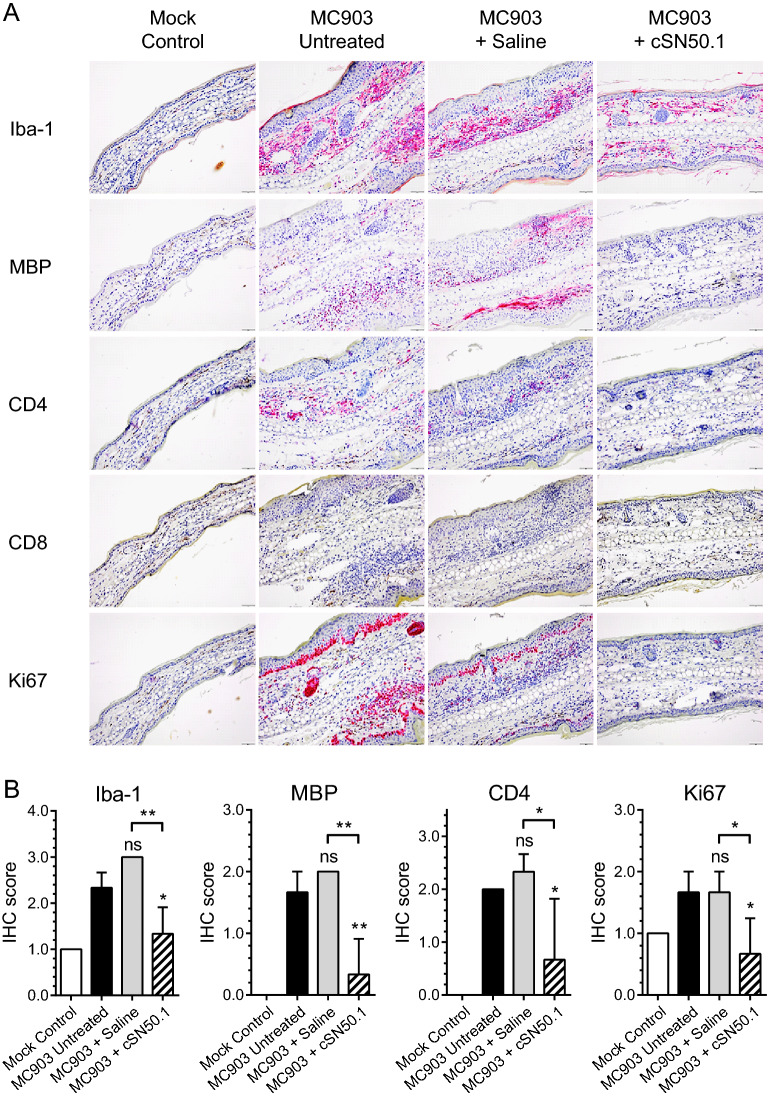
The attenuation of MC903-induced skin inflammation in the murine pinna by NTCI (cSN50.1 peptide) treatment. (**A**) Exposure to MC903 without treatment or treated with a saline control leads to cellular infiltration comprising predominately macrophages (Iba-1) and eosinophiles (major basic protein or MBP) combined with an influx of CD4 + T cells (CD4), leading to the thickening of the pinna as compared to the mock control. Moreover, the significant immunodetection of Ki67-positive cells indicated increased cellular proliferation in the basal cell layers of the epidermis in response to MC903^12^. Treatment with NTCI (cSN50.1 peptide) reduced the cellular mediators of the inflammatory response. NTCI reduced skin edema (see also Fig. 2A). Shown are representative images of pinna samples with immunohistochemistry for key inflammatory cell markers (× 20 magnification). (**B**) A semiquantitative analysis of immunohistochemical (IHC) staining presented in panel A. Scoring was conducted according to following formulas: IHC scoring (all inflammatory cell markers): 0—No or almost undetectable immunoreactivity (IR); 1—Scattered and rare IR in the dermis; 2—Multifocal and barely coalescing IR in the dermis; 3—Diffuse IR in the dermis. IHC scoring for Ki67 in epidermis: 0—None; 1—Single cell layer IR at basal zone of epidermis; 2—IR 2—5 layers thick within the basal zone of epidermis; 3—IR > 5 cell layers thick within the basal zone of epidermis. The data is presented as a mean + S.E.M. (n = 5). The statistical analysis was performed using ordinary one-way ANOVA with an uncorrected Fisher’s LSD test for a multiple comparison, *p < 0.05, **p < 0.005.

MC903-induced AD was manifested by infiltration of the skin with macrophages, eosinophils, and T lymphocytes, including CD4 + T cells. Inflammatory skin lesions consisted predominantly of macrophages and eosinophils, combined with an influx of CD4 + T cells, and significant proliferation of Ki67-positive cells in the basal cell layers of the epidermis (Fig. 4A). Immunohistochemical analysis also reflected NTCI control of the cellular effectors of AD. Thus, the major criteria for the experimental AD were controlled by NTCI. Consequently, we recorded a striking reduction of experimental AD manifested by redness, swelling, and infiltration of immune cells involved in skin inflammation.

Skin infiltration by eosinophils, macrophages, and T cells in response to MC903 was suppressed by NTCI treatment as documented by a semiquantitative analysis of immunohistochemical (IHC) staining (Fig. 4B). T cell infiltrates consisted primarily of CD4 + T cells with a paucity of CD8 + T cells. Strikingly, the proliferation of Ki67-positive cells in the basal zone of the epidermis was also attenuated. Therefore, we suggest that the cellular immunopathology of experimental AD is tightly linked to the genomic burst of the key mediators of skin inflammation. Taken together, these cellular mediators were suppressed by NTCI treatment thereby demonstrating a profound attenuation of the skin inflammatory response.

**Figure 5:**
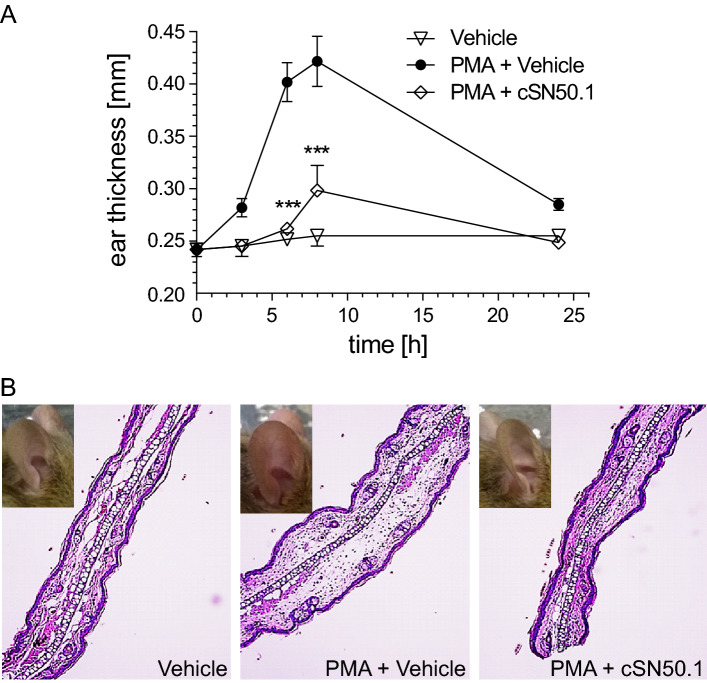
NTCI treatment reduces PMA-Induced ear inflammation. A single dose of Phorbol Myristate Acetate (PMA, 2 nmoles in 10 µl EtOH on each side) was administered topically to the right ear of 8-week-old female C57Bl/6 mice. Topical treatment with NTCI, cSN50.1 peptide (16.7 nmoles in 10 µl EtOH on each side), or vehicle (10 µl EtOH on each side) was administered 30 min before and 3, 6, and 8 h. after PMA challenge. A control group of mice was treated topically with vehicle only (10 µl EtOH) following the same treatment schedule. (**A**) Ear thickness was measured at 30 min before (0 h) and 3, 6, 8 and 24 h. after PMA challenge. Measurements are represented as mean ± S.E.M. (n = 5). Statistical significance of the difference between ear thickness in NTCI-treated mice (PMA + cSN50.1) and vehicle treated (PMA + Vehicle) or vehicle control (Vehicle) groups was determined by two-way ANOVA using corrected Holm-Sidak test for multiple comparison, ***p < 0.0005. (**B**) Ear images show increased swelling and redness induced by PMA (PMA + Vehicle). H & E staining of paraffin-embedded ear punch biopsies, collected 8 h. after PMA challenge, show increased swelling. Both signs of inflammation (redness and swelling) are reduced by NTCI treatment (PMA + cSN50.1). Displayed are representative images (× 20 magnification) of pinna sections.

Significantly, NTCI also suppressed skin inflammation induced by another agent, Phorbol-12-Myristate-13-Acetate (PMA), which produces severe thickening of the pinna (Fig. 5). Topically-applied PMA causes chronic skin inflammation mediated by Kit, a receptor tyrosine kinase required for mast cell accumulation^20^. The resolution of inflammation in this second model of experimental AD controlled by NTCI, is consistent with our prior discovery of its effectiveness in suppressing the PMA-induced nuclear transport of SRTFs comprising NF-κB, cFos/cJun, NFAT, and STAT1 in human T cells^21^. Thus, NTCI suppression of cardinal manifestations of MC903-induced AD (skin swelling, eczematous lesions, and dermal infiltrate), and PMA-induced skin inflammation attests to an effective and broad-range action of NTCI in these two preclinical settings.

## Discussion

We wish to report the NTCI, cell-penetrating cSN50.1 peptide, as a novel agent for treatment of AD, a recurrent inflammatory skin disease afflicting a sizable population of children and adults worldwide. A major step in understanding the mechanism of AD is the outcome of NTCI topical treatment of skin lesions caused by repeated challenge with MC903 (calcipotriol) for 23 days in an experimental model of AD. This vitamin D_3_ analog was found to induce the signs of AD during its clinical trial in patients with psoriasis^5^. Notably, TSLP-deficient mice did not develop AD when challenged with MC903 thereby assigning a major role to the gene encoding TSLP in the mechanism of AD^13^.

NTCI suppresses the gene encoding TSLP in mice challenged with MC903 (see Fig. 3), thereby providing a new line of evidence for the key role of TSLP in the mechanism of AD. Moreover, success of NTCI treatment also lies in the control of genes encoding other significant mediators of the T_H2_ response in AD, such as IL-4, its receptor, IL-4Rα, and IL-13. In addition, follicular helper T cells (T_FH_) are essential for humoral immunity and T-cell memory in atopic diseases^14^. The migration of T_FH_ cells depends on the chemokine receptor CXCR5 (CD185) regulated by SRTFs whose nuclear translocation is controlled by NTCI^7,22^. Furthermore, silencing of the gene encoding IL-23α, is significant as this cytokine induces AD-like inflammation in CCR2-deficient mice^23^. The gene encoding VEGF A, known as vascular permeability factor, was also suppressed by NTCI in the MC903-challenged skin explaining at least in part its reduction of swelling. Of note, the VEGF also contributes to the microvascular endothelial injury underling skin inflammation in psoriasis and other skin disorders^19^. Taken together, this outcome presents NTCI as a potentially effective and safe topical treatment of eczema and other inflammatory skin diseases.

Neuropathic itch is a significant sign of neurogenic inflammation in AD and other chronic skin inflammatory diseases^24,25^. In addition to triggering T_H2_-mediated allergic response, keratinocyte-derived TSLP activates cutaneous sensory neurons to induce itch^26^. Striking suppression of TSLP expression in the skin challenged by MC903 (see Fig. 3) explains the itch-reducing potential of NTCI. Moreover, its inhibition of nuclear translocation of STAT3 in human keratinocytes (unpublished results), and by extension, in sensory neurons, may also contribute to the itch-reducing action of NTCI. Both STAT3 and importin α5, which is the target of NTCI^9^, are involved in lesioned peripheral nerve, as reported by Ben-Yaakov and colleagues^27^.

The suppression of two other genes expressed in inflamed skin, IL-1β, and IL-6 (see Fig. 3) is also significant. These cytokines are responsible for the generalized inflammatory response known as the “acute phase protein response” associated with Endoplasmic Reticulum (ER) stress^7,28,29^. Thus, NTCI counteracts both the localized and, potentially, systemic inflammatory response in AD. Moreover, the uncontrolled action of IL-1β is linked to an inflammatory disorder of the skin and bones caused by the loss-of-function mutation of IL-1 receptor antagonist^30^. IL-1β also mediates ILC2-independent “spontaneous AD” in mice with defective skin barrier^31^.

**Figure 6:**
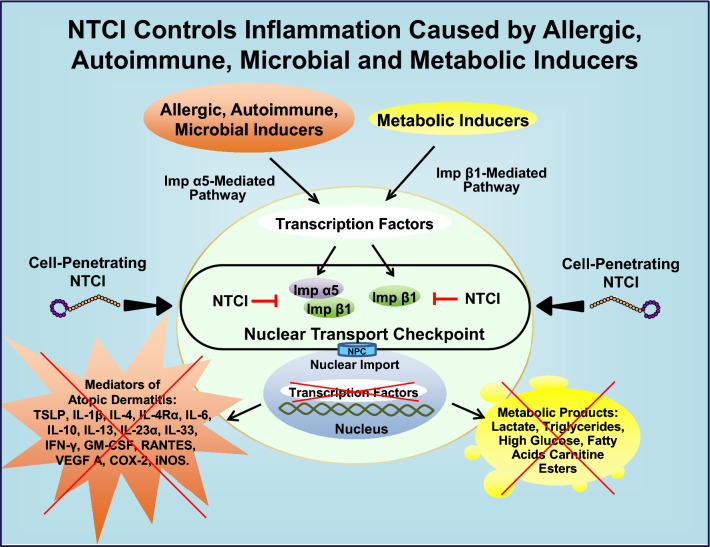
Mechanism of NTCI action in AD through targeted inhibition of importins α5 and β1 comprising the nuclear transport checkpoint. Proinflammatory Stress-Responsive Transcription Factors, which are activated by allergic, autoimmune, and microbial inducers of inflammation are ferried to the cell’s nucleus through an Importin α5-mediated pathway. Metabolic Transcription Factors, which are activated by overfeeding or inborn errors of metabolism are shuttled to the nucleus by Importin β1. NTCI stops binding of Transcription Factors to Importin α5 and Importin β1 thereby preventing the activation of the genes encoding mediators of Atopic Dermatitis as well as genes encoding metabolic intermediates. The latter underlie the link between AD and Metabolic Syndrome (see text and Suppl. Fig. S1 for expanded view of proinflammatory signaling to the nucleus comparing NTCI action with currently used drugs in AD). NPC, Nuclear Pore Complex.

The discovery of the nuclear transport checkpoint inhibitor’s role in the control of the key genes underlying the mechanism of AD is of particular significance. We submit that AD is chiefly mediated by the proinflammatory SRTFs (Fig. 6). These transcription factors encompass NF-ĸB. AP1 (cFos and cJun), STAT1, and NFATs^7^ as well as STAT3, the transcription factor involved in AD and other diseases mediated by allergic inflammation^32^.

In contrast to SRTFs and MTFs (MW > 45 kDa), the smaller transcription factors (MW < 45 kDa), some of which are essential to cell survival and maintenance, have “a free pass” to the nucleus, providing homeostatic control of the cell’s function and lifespan^33^. To this end, the smaller transcription factors cross the nuclear envelope while bypassing the nuclear transport checkpoint targeted by NTCI^10^. Accordingly, we found that NTCI treatment did not alter the expression of the five housekeeping genes (*Gusb, Hprt1, Hsp90ab1, Gapdh,* and *Actb*)^22^.

Our findings of significant suppression of the genes encoding TSLP and IL-1β have potential therapeutic implications for other human skin diseases caused by an inborn overproduction of these cytokines. The uncontrolled TSLP signaling plays a key role in patients with Netherton Syndrome, a rare hereditary skin disorder manifested by a susceptibility to AD, scaling skin, and hair abnormalities^34^. The uncontrolled action of IL-1β is linked to an inflammatory disorder of the skin and bones caused by the loss-of-function mutation of IL-1 receptor antagonist^30^. These and other autoinflammatory diseases of the skin and other organs are mediated by constitutive inflammation^7^. The afflicted patients may benefit from NTCI-based therapy suppressing TSLP, IL-1β, and other relevant and excessively acting mediators.

Clearly, the NTCI reported in this study represents a new class of broad-spectrum anti-inflammatory agents for topical (localized) and, if needed, systemic therapy. We are not aware of any published study of AD, which applied a similar method of controlling gene transcription by the inhibition of nuclear transport to suppress the expression of the key genes involved in the development of AD through the T_H2_ response. Among the other known broad-spectrum anti-inflammatory therapies, the glucocorticoids suppress inflammatory regulome via the action of their cognate nuclear receptor, which functions as transcription factor. However, glucocorticoids cause hyperglycemia, hyperlipidemia, osteoporosis, skin atrophy, and immunosuppression. In striking contrast, NTCI reduces blood glucose, cholesterol, and triglycerides, and increases the innate immunity-mediated clearance of bacteria^7^.

These points are relevant for the link between AD and microbial inflammation or metabolic inflammation^2^. First, AD predisposes skin to infections with *Staphylococcus aureus*^35^. The staphylococcal immunotoxin, Staphylococcal Enterotoxin B (SEB), also known as superantigen, stimulates IL-13 production, which is viewed as a potential etiologic factor in infants with AD^36^. NTCI suppressed the expression of IL-13 in the skin in our study (see Fig. 3). We anticipate, that NTCI would suppress staphylococcal immunotoxin-associated flare ups of AD consistent with our previous studies demonstrating NTCI control of SEB-induced toxic shock and acute respiratory distress syndrome^33,37,38^. Second, AD is associated with Metabolic Syndrome in pediatric and adult patients^39^. Its spectrum encompasses obesity, hyperglycemia, hyperlipidemia, fatty liver, atherosclerosis, and hypertension^40^. In the preclinical model of Metabolic Syndrome, due to the genetic ablation of the LDL receptor (*ldlr*^-/-^), combined with the Western Diet, NTCI controlled the metabolic hypersensitivity to a sublethal dose of LPS, a well-known inducer of microbial inflammation^17^. Thus, NTCI can also control AD, which is associated with inflammation caused by autoimmune, microbial, and metabolic inducers (see Fig. 6).

In summary, we successfully enabled NTCI to control the proinflammatory transcriptional mechanism of AD, the most common skin disease in the world. Our findings are of significant relevance to millions of individuals worldwide displaying signs of AD as well as other skin disorders that depend on the nuclear transport of transcription factors responding to inflammatory stress in human keratinocytes and immune cells. Our results provide a mechanistic blueprint for the translational application of NTCI (AMTX-100 CF), which is undergoing Phase 1/2 clinical trial for mild to moderate AD (NCT04313400).

## Materials and methods

### The synthesis and purification of cell-penetrating nuclear transport checkpoint inhibitor, cSN50.1 peptide

Our cell-penetrating NTCI peptide, cSN50.1 (AAVALLPAVLLALLAPCVQRKRQKLMPC, 2986 Da) was synthesized as described elsewhere^17^. Briefly, the peptide chain was assembled through Solid Phase Peptide Synthesis (SPPS) according to standard Fmoc chemistry protocols using an automated peptide synthesizer FOCUS XC (AAPPTec, Louisville, KY). Crude peptides were removed from the resin with a TFA cleavage cocktail and purified by dialysis against double-distilled water in 1 KDa membrane (Spectra/Por 7; Spectrum Laboratories, Rancho Dominguez, CA). The purity and structure of the final products were verified respectively by an analytical C18 RP HPLC (Beckman Coulter GOLD System, Brea, CA) and MALDI mass spectroscopy (Voyager Elite; PerSeptive Biosystems, Framingham, MA).

### Animal studies

Animal experiments were carried out in compliance with the ARRIVE guidelines and in strict accordance with the Guide for the Care and Use of Laboratory Animals of the US National Institutes of Health, and submitted protocols were approved by the Vanderbilt University Institutional Animal Care and Use Ethics Committee (Permit Number: A3227-01). Mice were closely monitored during the course of experiments and euthanized by Isoflurane inhalation followed by cervical dislocation at the experimental endpoint.

#### The experimental model of atopic dermatitis (Eczema)

Atopic dermatitis (AD)-like phenotype was induced in 8-week-old C57BL/6 female mice (The Jackson Laboratory) by topical application of the Vitamin D_3_ analog, calcipotriol (MC903)^11,41^. The experimental groups (Mock Control, n = 3; MC903 Untreated, n = 5; MC903 + Saline, n = 5; and MC903 + cSN50.1, n = 5) were selected using a double blinded randomization method^17^.

We used saline as a control representing a vehicle for all the NTCI peptide used in this study. This vehicle control is used in FDA-approved clinical studies of human subjects in which the tested new drug is administered, including therapeutic peptides. Each experiment was performed twice to assure statistical significance and experimental reproducibility.

The Treatment Protocol (see Fig. 1 for a graphic representation of the model and treatment protocol.): the initiation of AD-like phenotype induction began with a bolus dose of 6 nmoles MC903 (Millipore-Sigma #C4369) applied topically to the right ear (3 nmoles on each side). Repeated exposure to the irritant (once-a-day, Q.D.) was initiated on day 3 with 2 nmoles of MC903 (1 nmole on each side) and the challenge was continued for 21 days. NTCI treatment was introduced on day 20 as a twice daily (B.I.D.) topical application of its saline solution (10 µl of 10 mg/ml on each side) and was continued for 9 days. The control group of mice received a topical saline treatment (10 μl) on both sides of the right ear. On day 29, all mice were euthanized by over inhalation of isoflurane followed by cervical dislocation. 3–4 samples of the right ear (3 mm in diameter) were collected postmortem from auricula using a sterile disposable biopsy punch. One sample was fixed in 10% neutral-buffered formalin for histopathology and immunohistochemistry studies, remaining samples were snap frozen and stored at −80 °C for total RNA extraction.

#### PMA-induced skin inflammation

Skin inflammation was induced in 8-week-old C57BL/6 female mice (The Jackson Laboratory) by topical application of the Phorbol-12-Myristate-13-Acetate (PMA, Millipore-Sigma #524,400). The experimental groups (Vehicle, n = 5; PMA, n = 5; and PMA + cSN50.1, n = 5) were selected using a double blinded randomization method as described before^17^. A single dose of PMA (2 nmoles in 10 µl EtOH) was administered topically on each side to the right ear. Topical treatment with NTCI, cSN50.1 peptide (16.7 nmoles in 10 µl EtOH on each side of the right ear) or vehicle (10 µl EtOH on each side) was administered 30 min before and 3, 6, and 8 h. after PMA challenge. The unchallenged control group of mice was treated topically with vehicle only (10 µl EtOH) following the same treatment schedule. Ear thickness was measured at 30 min before (“0 h” timepoint) and 3, 6, 8 and 24 h. after PMA challenge. 3 mm ear punch biopsies were collected at 8 h. after PMA challenge from treated area under mild anesthesia (5% isoflurane). Ear samples were fixed in 10% neutral-buffered formalin for histopathology and immunohistochemistry studies. 24 h. after PMA challenge, all mice were, euthanized by over inhalation of isoflurane followed by cervical dislocation.

### Gene expression assay by real-time quantitative reverse transcription PCR

The mouse ear punch biopsies were disrupted in lysis buffer on ice using a Dounce hand homogenizer. Total RNA was isolated using NucleoSpin RNA Plus kit (Macherey–Nagel, Germany) according to the manufacturer’s instructions. RNA concentration and purity were determined using a NanoDrop One spectrophotometer (Thermo Fisher Scientific). 1 µg of the obtained RNA was reverse-transcribed using an iScript cDNA synthesis kit (Bio-Rad, CA). A real-time quantitative reverse transcription PCR was carried out in a 96-well plate on a QuantStudio 3 instrument using the Taqman Fast Advanced Master Mix (Thermo Fisher Scientific) according to the manufacturer’s protocol. The FAM-labeled probes for mouse genes were obtained from Thermo Fisher Scientific. The raw Ct values were converted into relative expression levels using Livak’s methods (2^−∆∆Ct^) with the 18S gene as a reference, and the Mock Control group as a calibrator (control). Converted Ct values were used for statistical analysis.

### Histology and immunohistochemistry

Pinna samples were collected by the postmortem punch biopsy of the skin (see above) and fixed overnight in 10% neutral buffered formalin, routinely processed, embedded in paraffin, sectioned at 5 μm and stained with Hematoxylin and Eosin (H&E). Immunohistochemistry analyses with antibodies against macrophages (Iba-1; FujiFilm, 019-19,741), eosinophils (MBP; Mayo Clinic, MR-14.7), CD4 (SYSY, HS-360 017), CD8 (SYSY HS-361 003), and Ki67 (Abcam, ab16667) were performed on the Leica Bond Max (Leica Biosystems Inc. Buffalo Grove, IL) following standard protocols in the Translational Pathology Shared Resource at Vanderbilt University Medical Center. Briefly, all assays were antigen retrieved using an Epitope Retrieval 2 solution for 20 min except MBP (Epitope Retrieval 1 solution for 15 min). Leica polymer was used as a secondary antibody for Iba-1, CD8, and Ki67 while a rat secondary antibody (Vector BA-4001) was used for MBP and CD4. The Bond Polymer Refine Detection system was used for visualization. Immunohistochemical stains were evaluated by a veterinary pathologist (K.N.G-C and K.L.B) blinded to the composition of the groups.

### Statistical analysis

Normal distribution of data sets was verified using normal probability plot (q-q) and Kolmogorov–Smirnov Normality Test. A statistical analysis was performed using tools built-in Prism 6 software (GraphPad). Gene expression profile in ear skin samples of C57Bl/6 mice as well as semiquantitative IHC scores were analyzed by ordinary one-way ANOVA with an uncorrected Fisher’s LSD test for a multiple comparison. The data is presented as a mean ± SEM. *p* values of < 0.05 were considered significant.

## Supplementary Information

Supplementary Information.

## Data Availability

All data generated or analyzed during this study are included in this published article and its supplementary information files.
