# Supplementary material for: Genomic control of inflammation in experimental atopic dermatitis
Source: Sci Rep. 2022 Nov 7;12:18891. doi: 10.1038/s41598-022-23042-x (PMC9640569; doi:10.1038/s41598-022-23042-x)
Supplement: Supplementary file 1 — Supplementary Information. [file 41598_2022_23042_MOESM1_ESM.pdf]

SUPPLEMENTARY MATERIALS

Genomic Control of Inflammation in Experimental Atopic Dermatitis

Yan Liu[1,2,#], Jozef Zienkiewicz[1,2,#], Huan Qiao[1], Katherine N. Gibson-Corley[3], Kelli L. Boyd[3], Ruth Ann Veach[4] and Jacek Hawiger[1,2,5,*]

[1] Vanderbilt University School of Medicine, Department of Medicine, Division of Allergy, Pulmonary and Critical Care Medicine, Nashville, Tennessee, United States of America

[2] Department of Veterans Affairs, Tennessee Valley Health Care System, Nashville, Tennessee, United States of America

[3] Vanderbilt University School of Medicine, Department of Pathology, Microbiology and Immunology, Nashville, Tennessee, United States of America

[4] Vanderbilt University School of Medicine, Department of Medicine, Division of Nephrology, Nashville, Tennessee, United States of America

[5] Vanderbilt University School of Medicine, Department of Molecular Physiology and Biophysics, Nashville, Tennessee, United States of America

[#] Y.L. and J.Z. contributed equally to this study.

* To whom correspondence should be addressed:
Jacek Hawiger
Vanderbilt University Medical Center
21st Avenue South, T-1218, MCN
Nashville, TN 37232, USA
phone: +1 (615) 828-8718
e-mail: jack.hawiger@vumc.org

Suppl. Fig. S1.

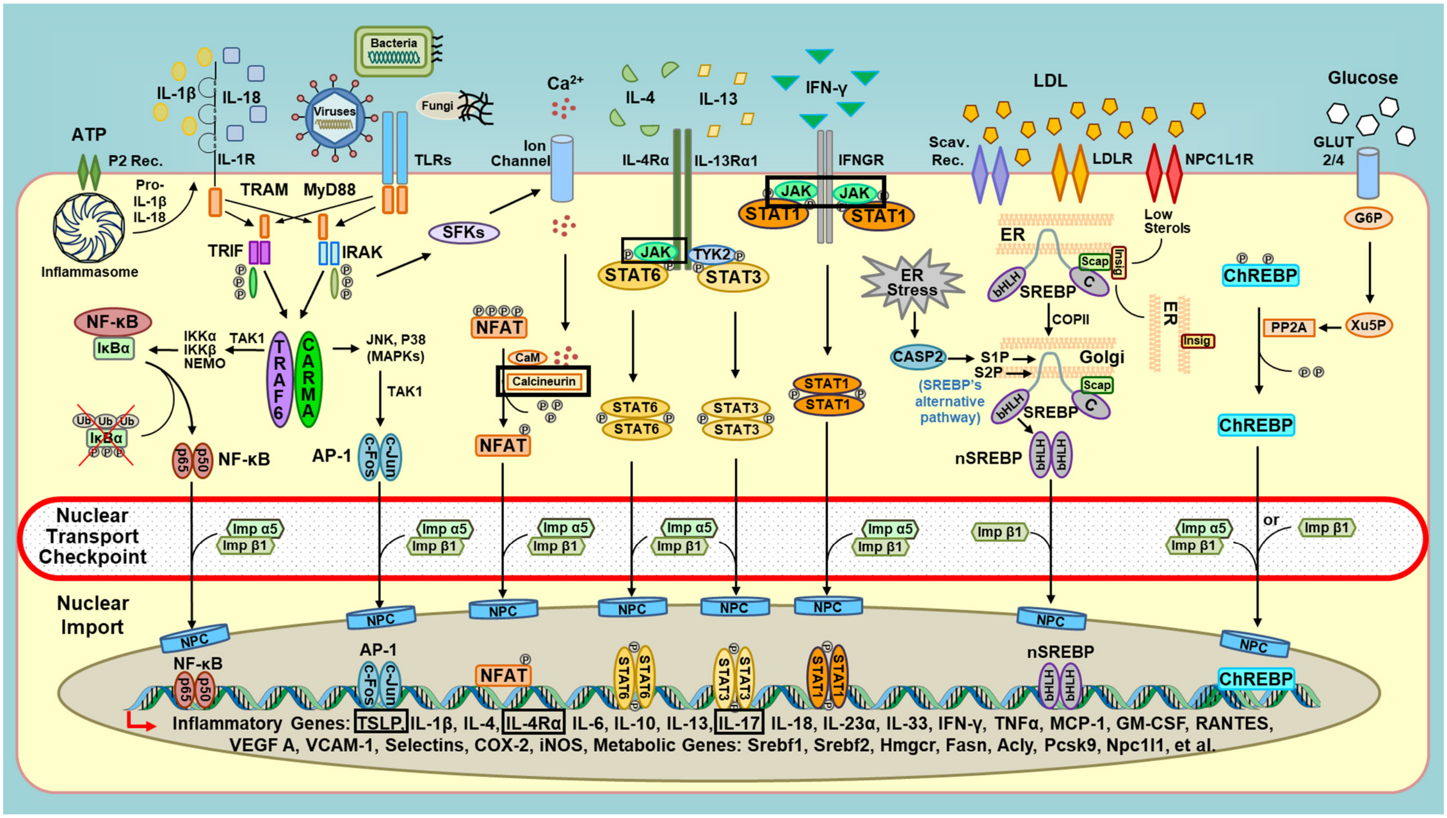

Inducers of Allergic, Autoimmune, Microbial, and Metabolic Inflammation Trigger Signaling Pathways that Mobilize Eight Families of Transcription Factors to Reach the Cell's Nucleus and Activate Multiple Genes Encoding Mediators of Inflammation. Nuclear Transport Checkpoint Inhibitor (NTCI) targets Importin α5 and Importin β1, which shuttle eight depicted transcription factor families thereby arresting their passage through the Nuclear Pore Complex (NPC). Hence, multiple inflammatory genes, that encode mediators of allergic, autoimmune, microbial, and metabolic inflammation, are suppressed by NTCI.
For comparison, the currently used anti-inflammatory drugs for treatment of Atopic Dermatitis target single inflammatory mediators depicted in black rectangles. Glucocorticoids are not displayed (see text for their mechanism of action).
